# Vaccination and monitoring strategies for epidemic prevention and detection in the Channel Island fox (*Urocyon littoralis*)

**DOI:** 10.1371/journal.pone.0232705

**Published:** 2020-05-18

**Authors:** Jessica N. Sanchez, Brian R. Hudgens

**Affiliations:** Institute for Wildlife Studies, Arcata, California, United States of America; Hiroshima University, JAPAN

## Abstract

Disease transmission and epidemic prevention are top conservation concerns for wildlife managers, especially for small, isolated populations. Previous studies have shown that the course of an epidemic within a heterogeneous host population is strongly influenced by whether pathogens are introduced to regions of relatively high or low host densities. This raises the question of how disease monitoring and vaccination programs are influenced by spatial heterogeneity in host distributions. We addressed this question by modeling vaccination and monitoring strategies for the Channel Island fox (*Urocyon littoralis*), which has a history of substantial population decline due to introduced disease. We simulated various strategies to detect and prevent epidemics of rabies and canine distemper using a spatially explicit model, which was parameterized from field studies. Increasing sentinel monitoring frequency, and to a lesser degree, the number of monitored sentinels from 50 to 150 radio collared animals, reduced the time to epidemic detection and percentage of the fox population infected at the time of detection for both pathogens. Fox density at the location of pathogen introduction had little influence on the time to detection, but a large influence on how many foxes had become infected by the detection day, especially when sentinels were monitored relatively infrequently. The efficacy of different vaccination strategies was heavily influenced by local host density at the site of pathogen entry. Generally, creating a vaccine firewall far away from the site of pathogen entry was the least effective strategy. A firewall close to the site of pathogen entry was generally more effective than a random distribution of vaccinated animals when pathogens entered regions of high host density, but not when pathogens entered regions of low host density. These results highlight the importance of considering host densities at likely locations of pathogen invasion when designing disease management plans.

## Introduction

Any given introduction of a novel pathogen into a fully susceptible host population has a wide range of possible outcomes [1,2]. When a pathogen is spread directly between hosts, depletion of local susceptible hosts can lead to rapid fadeout with few hosts suffering infection. This is possible even for diseases with a high epidemic potential (i.e., basic reproductive number, R_0_, >1) [1,3,4], and is more likely to occur when epidemics are restricted to areas of low host density [2]. Once an epidemic reaches a region of high host density or infects highly connected individuals (so-called “superspreaders”), rapid pathogen transmission becomes more likely and can lead to severe impacts on the host population [5,6]. Furthermore, high host density regions can conduct disease past low-density barriers because of snowball effects [2,7]. This often leads to a bimodal distribution of potential invasion outcomes which can be overlooked when focusing on the average course of epidemics, and traditional epidemic modeling approaches often fail to track the efficacy of disease monitoring or mitigation strategies [8].

Epidemic prevention strategies are most effective when they target the management of the most at-risk individuals or populations [6,9,10]. The success of these programs depends on accurately anticipating how a pathogen will spread among hosts [11,12]. However, species-specific data vital to modeling pathogen transmission in wildlife are often unavailable due to the difficulty associated with directly measuring variables such as host distribution, demographics, and behaviors [13]. Despite these data gaps, reducing host density has historically been one of the primary tools utilized to decrease host contact rates and transmission events [14,15], especially for directly transmitted pathogens with density-dependent transmission.

Commonly used management techniques to reduce the density of susceptible hosts include vaccination, culling, and fertility control. Natural epidemics and simulation models have demonstrated mixed efficacy of these techniques to prevent or minimize epidemics [11,16–18]. Culling often results in increased host movements and contact rates as surviving animals move into empty territories, and new residents immigrate into previously saturated habitats [18]. Fertility control reduces the number of new susceptible hosts entering the population in the form of new births [17], but has the potential consequence of reducing population size over time, which may not be acceptable for sensitive or threatened species. When available, vaccination is often the preferred option because it maintains a stable host population with an intact social structure, while effectively reducing the density of hosts susceptible to disease. Ideally, vaccines are distributed in ways that maximize the chances that an infectious host will contact a vaccinated (i.e. immune) host. These distribution strategies include the saturation of an exceptionally high risk “core area” with vaccinated animals, vaccination “firewalls” at the edge of an epidemic or in key geographic areas that act as a barrier to the advancing disease front, vaccinating key individuals that are highly connected with neighbors, or vaccinating animals randomly throughout the population [11,19,20].

The introduction of canine distemper virus (CDV) to an isolated population of immunologically naïve Channel Island foxes (*Urocyon littoralis*) demonstrates the significant and rapid impacts disease can have on a host population, and how important epidemic preparedness is for wildlife management. The island fox is endemic to six of the eight Channel Islands off the coast of southern California. Santa Catalina Island is geographically divided into eastern (~87% of the island’s land mass) and western sections by a narrow isthmus, which few foxes have been observed to cross [21]. The Catalina Island fox population was reduced by ~95% in less than one year following the introduction of canine distemper, probably by a mainland raccoon (*Procyon lotor*) that was a “stowaway” on a boat [21,22]. Luckily, the foxes inhabiting the smaller western portion of the island were largely untouched by the epidemic, likely because the narrow isthmus served as a barrier to fox movement and subsequent pathogen transmission to uninfected animals [21]. The speed and severity of this population decline, and the continued threat of future pathogen introduction from introduced species, resulted in the Santa Catalina Island fox subspecies being listed as “critically endangered” under the Endangered Species Act in 2004 [23].

The introduction of novel disease is a persistent risk to island foxes due to the regular movements of humans and animals (both purposeful and accidental) between the Channel Islands and the mainland. Implementation of a disease mitigation plan was a key part of the recent reclassification of the Santa Catalina Island fox subspecies to “threatened” and the delisting of three other subspecies formerly listed as "endangered" under the Endangered Species Act [24–27]. In an effort to prevent future epidemics, a subset of foxes has been vaccinated against CDV annually since 2000 [28] and against rabies annually since 2001 (personal communication, S. Timm 2012. E9731 County Road P, Westby, Wisconsin 54667). The Channel Island Fox Recovery Plan recommends vaccinating a minimum of 80–100 foxes against both rabies and CDV in two or three strategic geographic “core areas” on each island where there are relatively high fox densities and disease introduction risks, such as harbors where tourist and supply boats regularly dock [25,29]. These core areas could be clusters of vaccinated animals (e.g. in and around a town) or in the form of a “firewall,” where vaccines are distributed in a linear band in order to stop an advancing disease front [29]. Some islands (such as Santa Catalina) vaccinate a larger number of foxes and space these vaccinations randomly around the island because they have several areas at high risk for disease introduction [30].

The recovery plan also recommends the long-term monitoring of “sentinel” foxes [25]. “Sentinels” are radio-collared foxes that have not been vaccinated and are susceptible to introduced pathogens. They are monitored on a regular basis so managers will be alerted to an epidemic (or other population threat) when several sentinel animals die in quick succession or at a rate higher than the population’s baseline mortality. Sentinels also allow for quick carcass recovery, increasing the chances that a necropsy will identify a definitive cause of death.

Predictions of disease transmission in island foxes is complicated by sometimes drastic variation in fox density among different habitats within each island, resulting in similarly large variation in contact rates. Even across the relatively small spatial scale of an island, there is substantial variation in fox density across habitats within the same year [31] that can alter fox home ranges and contact rates [31,32]. Foxes have more neighbors with overlapping home ranges at higher densities, resulting in more total contact with other foxes [31] and potentially higher rates of pathogen transmission [2]. Spatial heterogeneity in fox density and contact rates results in the location of pathogen introduction having a strong influence on the potential outcome of an epidemic [2]. Therefore, the density of foxes at the site of pathogen introduction might also influence the efficacy of different monitoring and vaccination strategies.

Here, we evaluate the influence of fox density at the site of pathogen introduction on the efficacy of epidemic management strategies for San Clemente Island (SCI) foxes. Rabies and canine distemper are considered to be the two primary disease threats to island fox populations and are a focus of fox management activities on all of the California Channel Islands [25,29,33]. Both pathogens are directly transmitted and require close contact between hosts to spread. Rabies is most commonly transmitted through saliva when an infectious host bites a susceptible host [34]. CDV is generally transmitted through inhalation of aerosol droplets from the respiratory tract or contact with oral and ocular fluids of a sick animal [35,36]. We modified a previously published simulation model of rabies and CDV spread through SCI foxes [2] to evaluate the influence of host density on the success of monitoring and vaccination strategies for these two diseases. We used this model to answer three primary questions: 1) How does the number of collared sentinels and monitoring frequency affect the detection of an epidemic? 2) What is the optimal number and spatial distribution of vaccinated foxes to prevent an epidemic? 3) How does fox density at the site of pathogen introduction modulate the success of these monitoring and vaccination strategies?

## Methods

**Fig 1:**
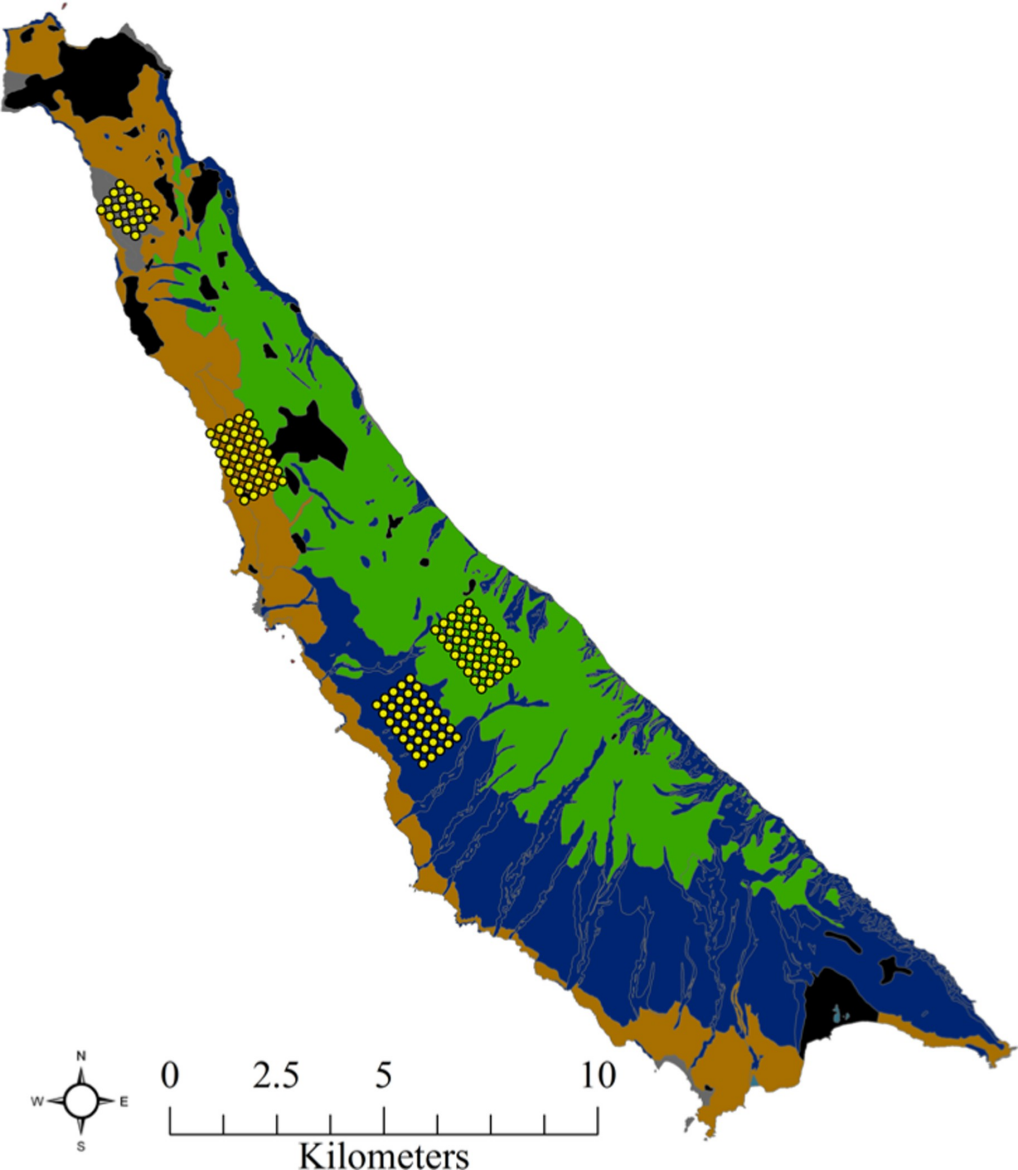
Map of San Clemente Island illustrating the four sites at which fox densities, home range size and overlap, and contact rates were measured. Yellow dots represent trapping grids where foxes were captured.

**Fig 2:**
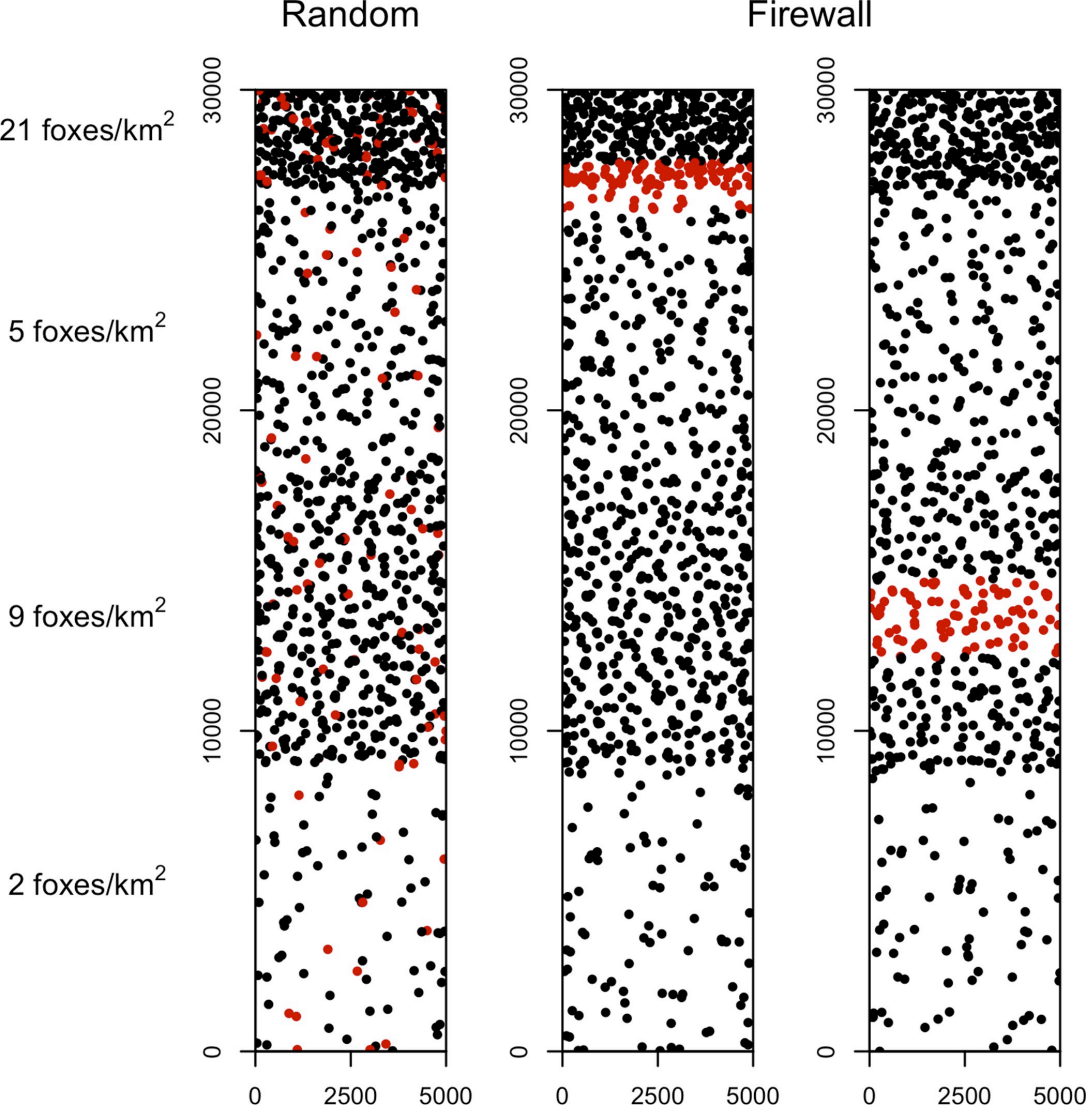
Maps of San Clemente Island as depicted by the rabies and canine distemper models, with dots representing the simulated home range center of each fox. Vaccinated individuals (red dots; 10% of the fox population) were distributed randomly across the island or in a firewall configuration located in a high or medium-high density of foxes. Fox densities within each section of the island represent the average across all model iterations. Scales are in meters.

We used Program R [37] to create a simplified island landscape to simulate the spread of infectious disease through a population of ~1,000 island foxes. We divided the island into four blocks, with each block reflecting the density of foxes observed at four study sites on San Clemente Island where fox home range sizes and contact rates had been previously measured (Fig 1) [31]. From north to south, the blocks were “high” density with 21 foxes/km^2^ representing sand dune habitat and developed areas, “medium-low” density with 5 foxes/km^2^ representing maritime desert scrub vegetation on gently sloping marine terraces, “medium-high” density with 9 foxes/km^2^ representing maritime desert scrub vegetation in rugged canyons and drainages, and “low” density with 2 foxes/km^2^ representing the grasslands dominating the central plateau extending the length of the island (Fig 2). The center points of simulated fox home ranges were placed at a random xy-coordinate on the island landscape, then a circular home range was generated based on the fox density block that the home range was centered in. The radius of each home range was sampled from a normal distribution derived from the observed relationship between fox density and home range size in SCI foxes (S1 Table) [2]. We sampled a daily contact rate for each pair of foxes from a normal distribution derived from the observed relationship between two-dimensional home range overlap [38] and contact rates for SCI foxes (S1 Table) [2,31]. The intercepts of both regressions were constrained to be nonnegative to reflect the impossibility of negative contact rates.

Simulated fox home ranges did not overlap more than 75%, which was the maximum degree of overlap observed between unrelated fox pairs on SCI [31]. Mates and family members (“related pairs”) have been shown to have greater home range overlap and contact rates than unrelated pairs [23,31,32,39]. The exclusion of related pairs simplifies the model since the proportion of neighboring foxes that are related is not known, but this assumption likely resulted in slower pathogen spread in the model simulations compared to what could be expected in reality.

The simulated fox population remained static with no births or sources of mortality except from the diseases being modeled. We adopted these simplifying assumptions to remove variables that would complicate our understanding of the role that vaccination and monitoring play in an epidemic. There is some biological justification for simplifying the model, which simulated host-disease dynamics for only a single year. Island foxes are seasonal breeders and pups are born in the spring [40], so we started the model right after pups were born and ended it right before the next birthing season began.

Every susceptible fox was given a small risk of contracting a pathogen from an infectious fox that did not occupy a neighboring home range in order to account for long-distance dispersal of juveniles, forays outside of established home ranges, and shifts in home ranges as foxes die from disease. This “background” transmission rate was based on the number of long-distance forays observed in SCI foxes and scaled with the number of infectious foxes in the population at each time-step (S1 Table) [2,41].

Each simulation was run for 365 daily time-steps. On the first day of the simulation, one fox was randomly selected at either the northern, high-density end of the island, or the southern, low-density end of the island. This fox was put into the infectious class to start the epidemic. At each time-step, the probability of infection for each susceptible fox was calculated based on the contact it had with neighboring infected foxes, the background transmission risk from all infected foxes across the island, and the transmission probability of the virus (S1 Table) [16,35,42–51]. Foxes moved between the susceptible, latent, infectious, or dead classes. Susceptible animals were disease free and unvaccinated, and therefore capable of becoming infected with a pathogen. Latent animals had been infected but were not yet capable of transmitting the pathogen to other foxes and remained in this class for an average of 42 days (maximum 90 days) for rabies and an average of 5 days (maximum 14 days) for CDV (S1 Table). Infectious foxes were capable of transmitting a pathogen to susceptible foxes and remained in this class for an average of 4 days (maximum 14 days) for rabies and an average of 21 days (maximum 60 days) for CDV (S1 Table). All infected animals eventually died.

Rabies is almost always fatal to mammals, and data on recovery is incomplete [52,53] so a recovered class was not included for this disease. Recovery of island foxes from CDV infection is likely very rare, based on the high mortality observed during the Santa Catalina Island CDV epidemic [21] and in other *Urocyon* species [36,54,55]. Previous sensitivity analyses for this model included CDV recovery and found that it increased pathogen transmission and decreased the probability of epidemic fadeout due to the extended shedding period of recovering animals, and these changes were dependent on the percentage of foxes recovering from infection [2]. Recovery was not included in the model treatments presented here because island fox recovery rates are unknown and the focus of this manuscript is on evaluating monitoring and vaccination tools.

We used the simulation to evaluate two aspects of monitoring effort: monitoring intensity (i.e. number of sentinels monitored) and monitoring frequency (i.e. how often the mortality status of sentinels was determined). The level of monitoring intensity was simulated by randomly selecting 50, 75, 100, 125 or 150 foxes to be sentinel animals, representing approximately 5–15% of the total fox population. When a sentinel died from disease, we recorded its day of death and the number of foxes in the population that were infected (including the latent, infectious, and dead classes) on that day. Most monitoring schemes currently used on the California Channel Islands check the status of sentinels weekly. This means that a carcass will be a minimum of 1–7 days old before it is detected. Depending on ambient environmental conditions, tissues can rapidly decay beyond the point where a necropsy can identify the cause of death even when carcasses are collected quickly following a status check revealing a mortality. Consequently, it is not unreasonable that multiple sentinels could perish from a disease before the discovery of a burgeoning epidemic. Epidemic response plans in place for San Clemente Island, Catalina Island, and the Channel Islands National Park call for increased monitoring frequency if more than 2–3 sentinels (depending on the number of sentinels monitored) die within a 30 day period, regardless of cause [26,27]. To account for these potential delays in the identification of disease and elevation in mortality rates, we assume that under typical monitoring frequencies, an epidemic is not confirmed until the fifth sentinel has died. This corresponds to an expected 80% of sentinel carcasses being too far decomposed to determine the cause of death, similar to the delay in detection estimated by Doak et al. [12]. We also evaluated the maximum possible benefits of increasing monitoring frequency by assuming a high-frequency monitoring strategy sufficient to confirm an epidemic with the first sentinel mortality.

Model output for sentinel monitoring treatments included the number of days between when the first fox was infected and the epidemic was detected ("detection day") and the percentage of the total fox population that was infected on the detection day. Monitoring effort treatments assumed that no animals were vaccinated.

We also evaluated two aspects of vaccination strategy: the percentage of the population vaccinated and the distribution of vaccinated animals on the landscape. Vaccination simulations included four levels of vaccination: 0%, 10%, 30%, or 50% of the fox population. The target annual vaccination rate on SCI is 10%, and 30% approximates the highest vaccination rate achieved on any of the Channel Islands [33]. A 50% vaccination rate has been recommended by island managers as a worthwhile target if it is effective in stopping an epidemic [30].

At each level of vaccination, we modeled the two vaccination distribution strategies currently implemented on the Channel Islands [29,30]. Vaccinated animals were distributed at random across the island or grouped together in a firewall [29] that spanned the entire width of the island (Fig 2). The location of the vaccination firewall varied based on the simulated disease introduction site. In simulations of an epidemic originating in the high-density, northern portion of the island (e.g., landing docks or the town), the vaccination firewall was placed at the border between the high and medium-low density habitats (Fig 2). In simulations of an epidemic originating in the low-density, remote southern beaches, the vaccination firewall was placed in the medium-high density block (Fig 2). This represents an optimistic assumption that the most likely site of disease introduction can be predicted. However, complete saturation of the local fox population with vaccines is not feasible, so there will always be some number of susceptible foxes surrounding the first infected fox even when the introduction site is correctly anticipated. The more foxes that become infected before the disease front reaches the vaccine firewall, the greater the risk that one of these infected foxes will “jump” the firewall through a long-distance dispersal event, abnormal movements of sick animals, or home range shifts as foxes die and habitat is left unoccupied (simulated by the “background” transmission rate). On the other hand, placing a firewall closer to the anticipated point of pathogen introduction carries a greater risk if introduction occurs on the unanticipated side, leaving fewer animals protected. To evaluate the importance of firewall location relative to the location of pathogen introduction, we tested two treatments for both low and high-density firewalls. Each firewall was placed so that either ~20% (200 foxes; “far firewall” treatments) or ~5% (50 foxes; “near firewall” treatments) of the total fox population was left on the “infected” side of the firewall where a pathogen was introduced. These treatments represent a range of scenarios that allowed us to explore the importance of predicting disease entry points on the efficacy of a vaccination firewall.

Model output for vaccination treatments included the percentage of iterations resulting in epidemic fadeout (no latent or infected animals remaining at the end of the simulation), and the percentage of the total fox population that was infected at the end of the simulation (day 365).

Vaccinated foxes were placed in the vaccinated class on the first day of the simulation and were never susceptible to infection. We assumed vaccinations provided 100% immunity that lasted for the entire simulation. This is a reasonable assumption for rabies; dogs have been found to have 100% immunity after 2–4 doses of vaccine, with antibody levels suggesting that one dose is adequate [56]. However, only 50%– 83% of Siberian polecats (*Mustela eversmanni*) injected with CDV vaccine may develop immunity (two doses delivered four weeks apart) [57]. In order to achieve the target number of immune animals, managers may want to vaccinate 20%– 50% more animals than they hope to ultimately have protected and recapture these animals to give them booster vaccinations to maintain immunity. The model assumed that this has been done, and vaccinated animals represent individuals who were completely protected from infection.

We ran 1,000 iterations for each of the model treatments testing monitoring strategies (five levels of monitoring intensity and two levels of monitoring frequency for two introduction sites and two pathogens) and model treatments testing vaccination strategies (three levels of vaccination with three distribution strategies for two introduction sites and two pathogens). For each model treatment, we recorded the median and 25% and 75% quartiles (Q1 and Q3, respectively) of each output variable. Medians and interquartile ranges (IQRs) were chosen to summarize the data because the distributions of output variables were often highly skewed.

## Results

### Sentinel monitoring

**Fig 3:**
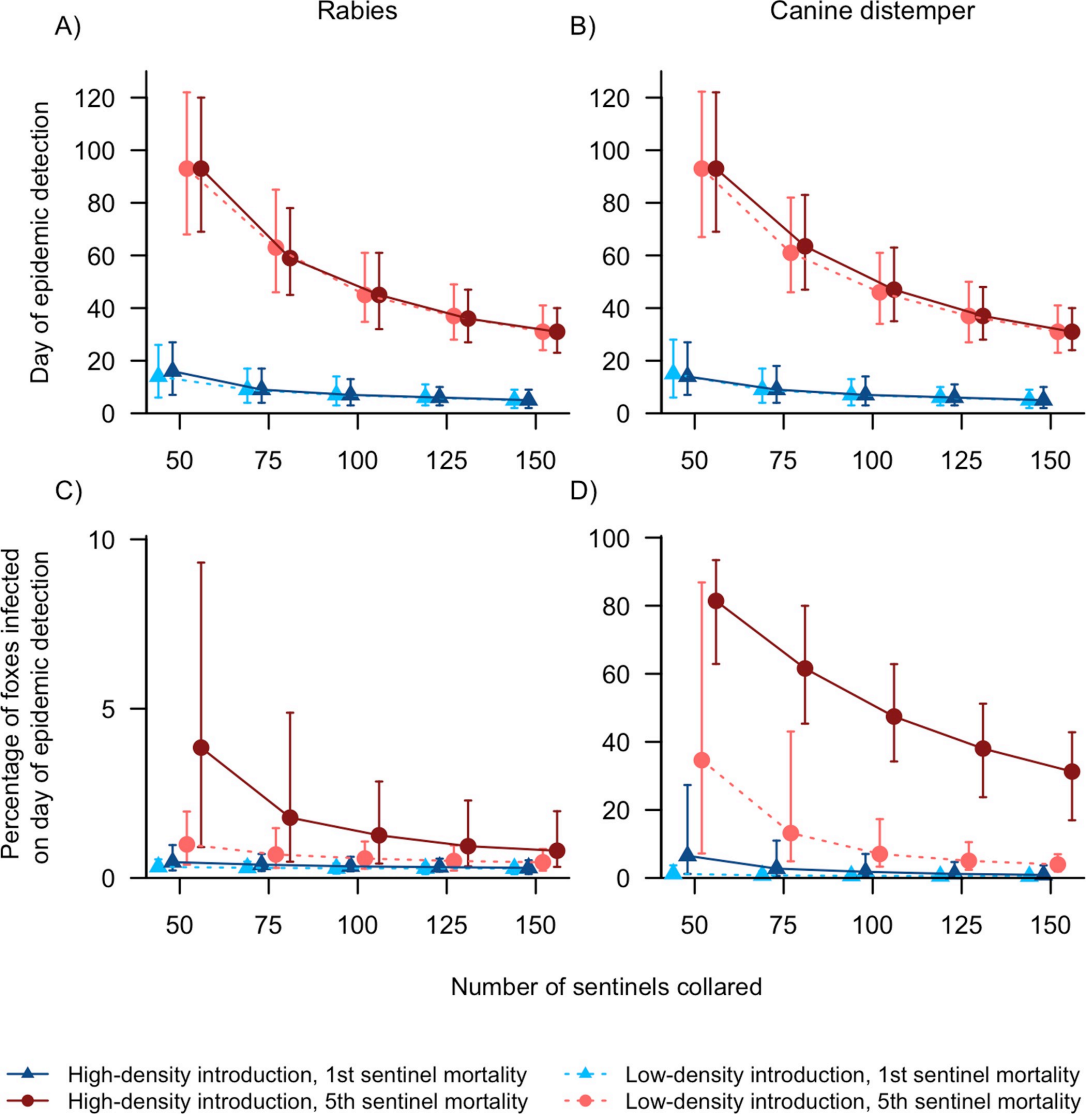
Model simulation results (medians, 25% and 75% quartiles) of rabies (A, C) and canine distemper (B, D) introduction to areas of San Clemente Island with low or high fox density and no vaccination, with varying numbers of radio-collared sentinels. As the number of unvaccinated sentinel foxes increased, the number of days until epidemic detection (A, B) and the percentage of foxes infected (i.e. latent, infectious, or dead) by the day of detection (C, D) decreased. Note the different y-axis of panel C.

Monitoring frequency had the single biggest impact on the time between pathogen introduction and epidemic detection. When monitoring frequency was sufficient to confirm an epidemic from the first sentinel death, epidemics were detected within a median of 5–16 days regardless of pathogen, introduction site, or monitoring intensity (Fig 3A and 3B and S2 and S3 Tables). When monitoring frequency was lower (epidemic detection occurred from the fifth sentinel death), monitoring intensity had a larger influence on time to detection, which declined from a median value of 93 days with 50 sentinels to 31 days with 150 sentinels (Fig 3A and 3B and S2 and S3 Tables). Pathogen and introduction site had little influence.

The variability in time to epidemic detection among model iterations (error bars in Fig 3A and 3B) was influenced by monitoring frequency and intensity. When only 50 sentinels were monitored, the IQR of detection day was 51–55 days with low-frequency monitoring and 20–22 days with high-frequency monitoring (Fig 3A and 3B and S2 and S3 Tables). Increasing the number of sentinels to 150 reduced the IQR to 16–18 with low-frequency monitoring and 7–8 days with high-frequency monitoring (Fig 3A and 3B and S2 and S3 Tables). Overall, increasing monitoring frequency led to greater confidence that epidemics would be detected within three weeks of initial infection. Increasing monitoring intensity by collaring more sentinels also reduced model variability, but to a lesser extent.

The benefits of early detection, in terms of how many foxes remained uninfected by the time an epidemic was discovered, was influenced by both pathogen and introduction site. In the case of rabies, which tends to spread relatively slowly [30], a median of <4% of the population was infected by the day of detection regardless of monitoring strategy or pathogen introduction site (Fig 3C and S2 Table).

In contrast to rabies, there was a wide range of potential outcomes with a CDV epidemic in terms of the percent of foxes infected at epidemic detection (Fig 3D and S3 Table). The benefits of increased monitoring effort largely depended on pathogen introduction site. Increasing the number of sentinels yielded larger benefits when the initial CDV infection was in the high fox density region than when it was in the low fox density region. This was most dramatic in the worst case scenario, 50 sentinels monitored and confirmation at the fifth mortality, which resulted in 81.4% foxes becoming infected when an epidemic started in the high fox density region, compared to 34.6% of foxes when the epidemic started in the low density region (Fig 3D and S3 Table). However, there was high variability in model outcomes (as indicated by the wide IQR), especially with low-density introduction (Fig 3D and S3 Table). Overall, increased monitoring frequency paid bigger dividends than increased numbers of sentinels. At the first sentinel mortality, a median of <10% of foxes were infected at the time of epidemic discovery regardless of pathogen introduction site or number of sentinels monitored (Fig 3D and S3 Table). There were still consistent declines in the percentage of foxes infected as the number of sentinels increased, but these were negligible compared to the reduction between the first and fifth sentinel mortality (Fig 3D and S3 Table).

Increasing the number of sentinels and the monitoring frequency also reduced the variability in the percentage of foxes infected at the end of one year, but these improvements were most drastic for CDV. For rabies, the IQR was always narrow, with the largest IQR occurring with high-density introduction, low-frequency monitoring, and only 50 sentinels (IQR = 8.4%; Fig 3C and S2 Table). All other rabies treatments had IQRs <4.4% (Fig 3C and S2 Table). For CDV, increasing the number of sentinels generally reduced the IQR across all combinations of introduction site and monitoring frequency. The most drastic reductions were for low-density introduction sites with low-frequency monitoring, where the IQR shrunk from 79.6% with 50 sentinels to 5.1% with 150 sentinels (Fig 3D and S3 Table). Increasing monitoring frequency also reduced outcome variability, especially for low-density introduction treatments. When only 50 sentinels were monitored, the IQR of foxes infected was 3.3% when the first sentinel was detected, compared to 79.6% when the fifth sentinel was detected (Fig 3D and S3 Table). For both pathogens, the decrease in model variability was generally largest between 50 and 100 collared sentinels, with the incremental improvements decreasing as the number of sentinels increased between 100 and 150 animals (Fig 3 and S2 and S3 Tables).

### Vaccination

#### Rabies

**Fig 4:**
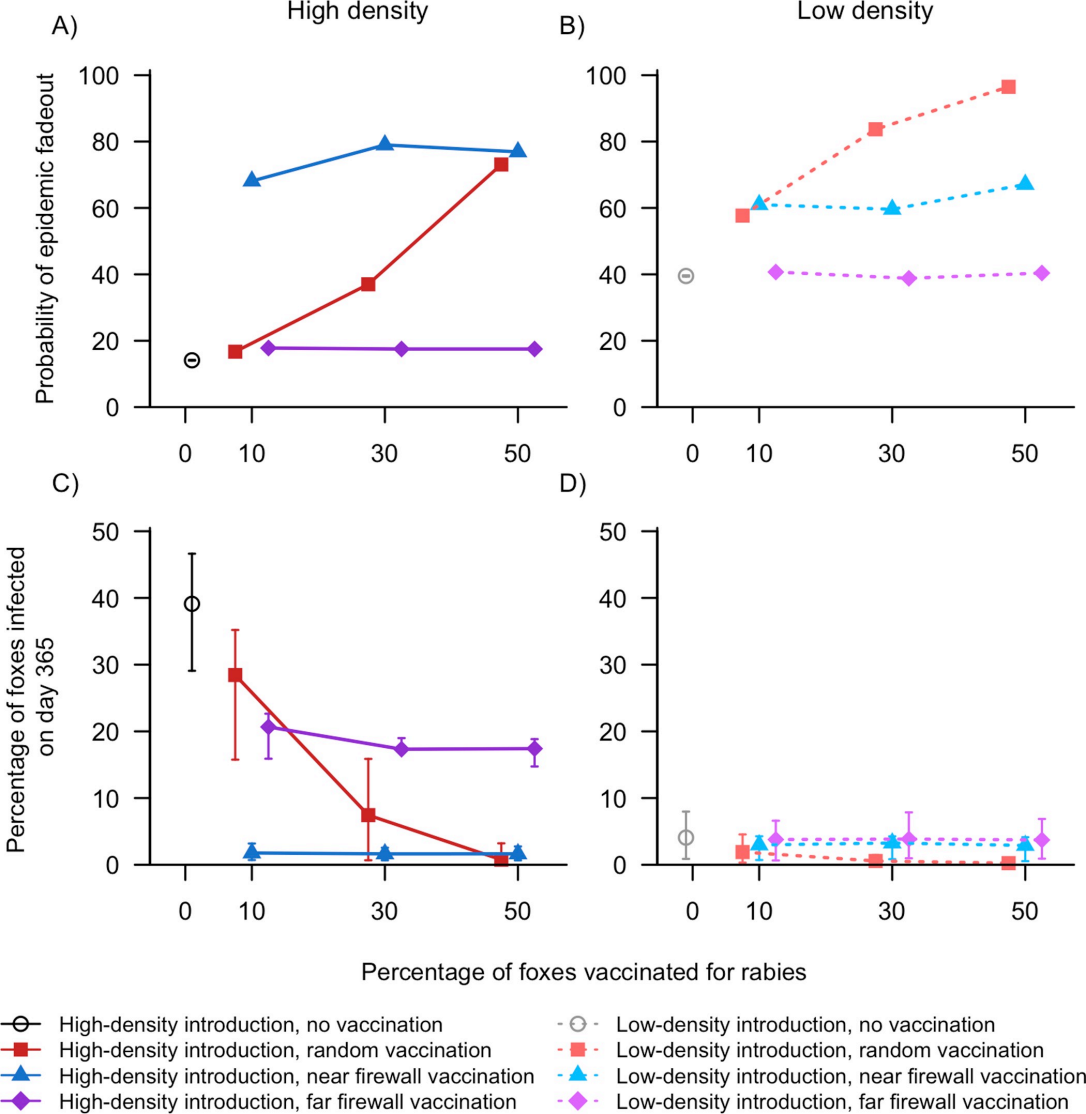
Model simulation results (medians, 25% and 75% quartiles) of rabies introduction to areas of San Clemente Island with high (A, C) or low (B, D) fox density and varying levels of fox vaccination (0%, 10%, 30%, or 50% of the population) distributed randomly or in a firewall. Simulations with firewall vaccination had either 5% of foxes (“near firewall”) or 20% of foxes (“far firewall”) on the side of the firewall where rabies was introduced. As the percentage of vaccinated foxes increased, the probability of epidemic fadeout (A, B) generally increased and the percentage of foxes infected (i.e. latent, infectious, or dead) by the end of the 365-day simulation (C, D) generally decreased. Note differing y-axes between upper and lower panels.

The probability that a rabies epidemic would fadeout within one year increased with random vaccination and near firewall vaccination compared to no vaccination, but not with far firewall vaccination (Fig 4A and 4B and S4 Table). The probability of fadeout with random vaccination increased substantially as the percentage of foxes vaccinated increased (Fig 4A and 4B and S4 Table). However, both firewall treatments had <11% increase in fadeout probability with increased vaccination rates (Fig 4A and 4B and S4 Table). At high fox densities, near firewall was more effective than random vaccination, with the gap between the two treatments shrinking as more foxes were vaccinated (Fig 4A and S4 Table). An opposite effect was observed at low fox densities, where random vaccination was more effective than near firewall vaccination and the gap between the two treatments increased as more foxes were vaccinated (Fig 4B and S4 Table). Most model treatments had a large percentage of iterations in which rabies did not fadeout and was continuing to spread slowly through the population at day 365.

All vaccine treatments reduced the percentage of foxes infected at the end of one year compared to no vaccination. At high fox densities, near firewall vaccination was the most successful strategy, with a median of <2.0% infected foxes regardless of vaccination level (Figs 4C and S1C and S4 Table). Far firewall vaccination reduced the percentage of infected foxes compared to no vaccination by ~20%, but never resulted in <17% of the population infected even with the highest level of vaccination (Figs 4C and S1E and S4 Table). Both firewall treatments produced minimal additional reductions in infected foxes with increasing levels of vaccination. Increasing levels of randomly placed vaccinations led to decreases in the percentage of infected foxes, with the largest decrease occurring between 10% and 30% (Figs 4C and S1A and S4 Table). At 10% vaccination, random vaccination was comparable to far firewall vaccination but resulted in 26.7% more infected foxes than near firewall vaccination. At 50% vaccination, random vaccination was comparable to near firewall vaccination and resulted in 16.7% fewer infected foxes than far firewall vaccination (Fig 4C and S4 Table).

At low fox densities, both firewall treatments were unaffected by the level of vaccination, but random vaccination led to some decline in infected foxes as vaccination levels increased (Fig 4D and S4 Table and S1B, S1D and S1F Fig). However, these declines were negligible, with a median of <4.0% of foxes becoming infected in all low-density vaccination treatments.

Model outcome variability was similar across vaccination levels for most treatments, although there was substantial reduction in variability between 30% and 50% for random vaccination at high densities (Figs 4C and 4D and S1 and S4 Table).

#### Canine distemper

**Fig 5:**
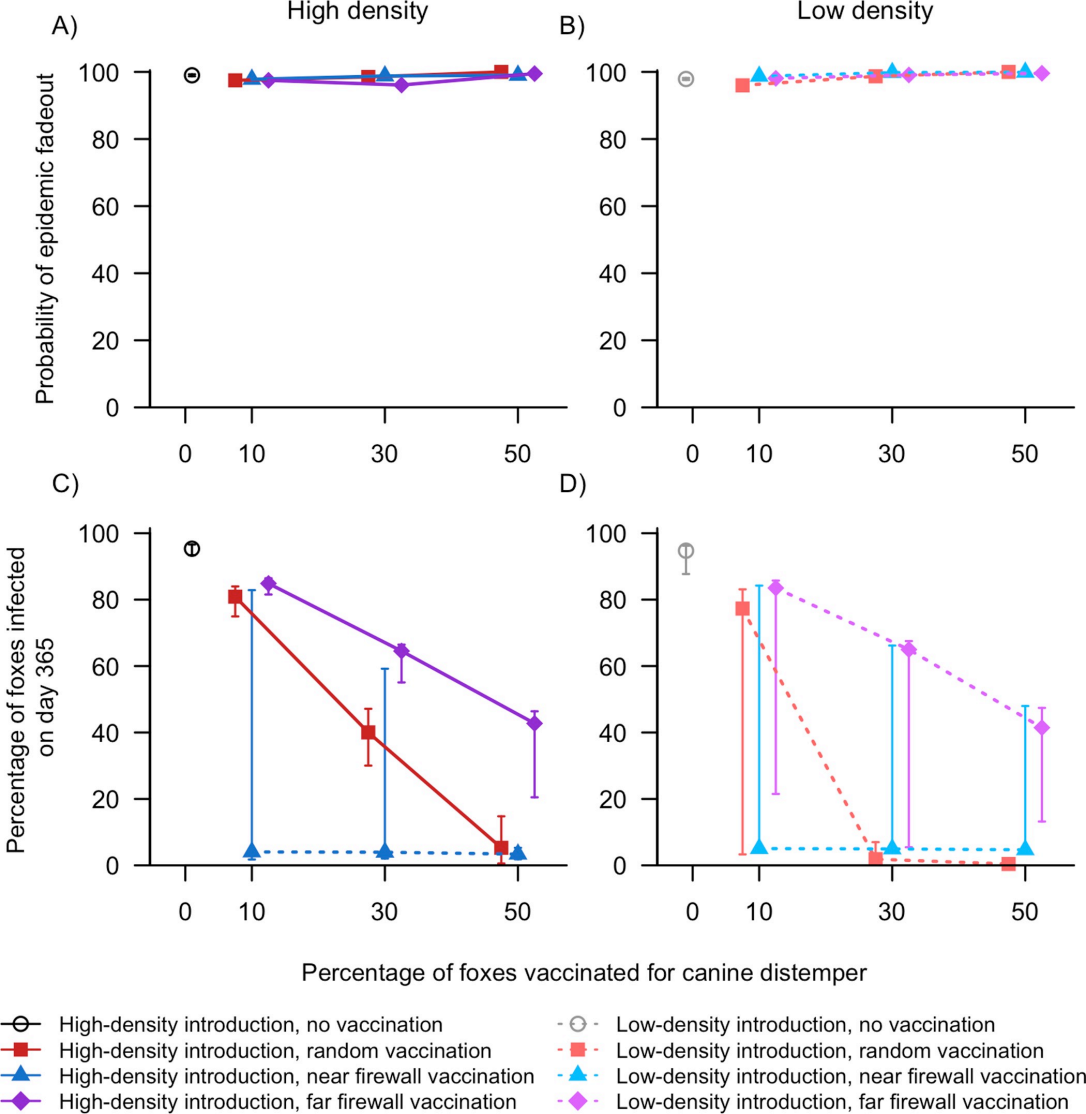
Model simulation results (medians, 25% and 75% quartiles) of canine distemper introduction to areas of San Clemente Island with high (A, C) or low (B, D) fox density and varying levels of fox vaccination (0%, 10%, 30%, or 50% of the population) distributed randomly or in a firewall. Simulations with firewall vaccination had either 5% of foxes (“near firewall”) or 20% of foxes (“far firewall”) on the side of the firewall where canine distemper was introduced. As the percentage of vaccinated foxes increased, the probability of epidemic fadeout (A, B) generally remained stable and the percentage of foxes infected (i.e. latent, infectious, or dead) by the end of the 365-day simulation (C, D) generally decreased.

For CDV, the probability of epidemic fadeout was always very high, with medians >96% across all vaccination levels, vaccination distributions, and fox densities (Fig 5A and 5B and S5 Table). Depending on the model treatment, fadeout occurred due to a lack of susceptible foxes either locally (with minimal fox infections) or after the disease spread through the entire fox population (infecting most foxes on the island). This bimodal distribution of model outcomes is reflected in the large IQRs in the percentage of infected foxes (Figs 5C and 5D and S2 and S5 Table). Many model treatments have large IQRs spanning almost the entire range of susceptible foxes (ex. 0 − 90% of foxes infected with 10% vaccination) while the median values tend to be extremely high or very low (Fig 5C and 5D and S5 Table).

All vaccine treatments reduced the percentage of foxes infected at the end of one year compared to no vaccination, but there were dramatic differences in the efficacy of each treatment. At high fox densities, both random and far firewall vaccination treatments resulted in a near linear decrease in infected foxes with increasing levels of vaccination (Figs 5C and S2 and S5 Table). For far firewall vaccination treatments, the median percentage of foxes infected was within 5–7% of the total susceptible (i.e. unvaccinated) population, demonstrating that nearly the entire unvaccinated population had become infected by the end of one year (ex. a maximum of 70% of foxes can become infected with 30% vaccination; Figs 5C and S2E and S5 Table). However, variability in model outcome increased as vaccination levels increased, reflecting that higher vaccination rates did result in more model iterations where larger numbers of susceptible foxes survived. In contrast, for random vaccination placement, increasing vaccination levels led to greater protection for the remaining unvaccinated foxes without leading to greater variability in model outcome (Fig 5C and S5 Table and S2A Fig). For example, at 50% vaccination rates, a median of 5.3% out of a possible 50% of foxes were infected at the end of the simulation (Fig 5C and S5 Table and S2A Fig). Near firewall vaccination was by far the most successful vaccine distribution, with a median of ≤4.0% of foxes infected across all levels of vaccination (Fig 5C and S5 Table and S2C Fig). However, there was high model variability when only 10% or 30% of foxes were vaccinated (IQRs of 81.1% and 57.2%, respectively; Fig 5C and S5 Table and S2C Fig). The upper quartiles of infected foxes came within 3.6% of the maximum susceptible foxes available, meaning there were 25% of model iterations in which all unvaccinated foxes became infected (S2C Fig). This reflects that while near firewall vaccination was an effective strategy most of the time, if the firewall was breached then CDV infected the entire fox population within one year.

At low fox densities, the efficacy of different vaccination strategies generally mirrored the high-introduction site scenarios (especially for near firewall treatments), though there was greater variability in model outcomes. Far firewall vaccination had a high median of infected foxes similar to with high-density introduction, but the IQRs were wider and skewed towards very low percentages of infected foxes. This shows there were more iterations in which CDV faded out before many unvaccinated foxes were infected (Fig 5D and S5 Table and S2F Fig). Random vaccination had a high median of infected foxes with just 10% vaccination, but the IQR was much wider than at high densities and skewed towards smaller percentages of infected foxes, again reflecting that sometimes this strategy was highly effective in stopping the epidemic (Fig 5D and S5 Table and S2B Fig). Random vaccination was much more effective at 30% vaccination with low-density introduction, where a median of <2.0% of foxes became infected with CDV.

## Discussion

When a disease management strategy is primarily reactive to the detection of infected individuals, monitoring effort plays an important role in reducing the severity of epidemic impact on host populations [58]. However, while monitoring radio-collared sentinels is a commonly recommended component of epidemic mitigation, little research has been done on optimal monitoring strategies. Our model showed that more monitoring effort, both in terms of increasing the number of sentinels monitored and monitoring frequency, always led to earlier epidemic detection and fewer infected foxes on the day of detection. However, realistic budget constraints require that choices be made about how many foxes are monitored and at what frequency, with increases in one generally leaving fewer resources available for the other. Our findings corroborate conclusions from nonspatial models of disease spread in island foxes that reducing the time between sentinel mortality and implementation of epidemic management actions is more effective than increasing the number of sentinels in a population [12]. These results are driven by the fact that early in the course of an epidemic the rate at which susceptible foxes become infected is increasing more rapidly than the rate at which infected animals are dying, with the number of new infections per day peaking between the median detection date of the first and fifth sentinel mortality [2,12]. The cost of delays in epidemic detection or management response time will be greatest for pathogens capable of rapid initial spread. For example, differences in the number of infected animals at the time of the first vs. fifth sentinel mortality were greater for CDV than for rabies, and greater for epidemics beginning in regions of high fox density than those beginning in regions of low fox density (Fig 3 and S2 Table).

Our results from both pathogens show there is a relationship between the spatial distribution of vaccines and the level of vaccination required to stop pathogen transmission, and this relationship is dependent on the heterogeneity of host densities. In low host density regions, vaccinating randomly has a greater potential to suppress susceptible host densities below the epidemic threshold (R_0_ < 1) and prevent an epidemic from ever establishing. When a pathogen invades a region with high host densities, the same proportional vaccination effort is less likely to reduce the local density of susceptible individuals below the epidemic threshold [59] and a firewall close to the introduction site may be more effective. Similar work has shown the complexity of this relationship, with some studies showing that vaccine firewalls effectively reduced the spatial spread and impact on host populations for a wide range of diseases [10,11], and others showing that spatial aggregation of vaccinated animals increases the proportion of animals that must be vaccinated to cause epidemic fadeout [60,61]

We reaffirmed findings from previous simulations that showed vaccination levels substantially lower than 70% are sufficient to confer herd immunity to rabies in spatially structured populations [62–65]. The minimum vaccination level needed to protect a target proportion of hosts depends on both the distribution of vaccinated animals and overall host density. Random vaccination rates continued to improve rabies epidemic outcomes as the level of vaccination increased, even up to 50% vaccination. In contrast, when a vaccine firewall was placed very close to the site of pathogen introduction, vaccination rates as low as 10% were sufficient to minimize the number of infected foxes and cause epidemic fadeout in >60% of model iterations. However, rabies had a greater impact on the fox population when it was introduced to high-density regions or when vaccinations were distributed in a firewall far away from the site of pathogen introduction. Consequently, even though vaccination protects some fraction of the unvaccinated population through herd immunity during first year of an epidemic, vaccination rates <50% should not be relied upon to prevent rabies from persisting in the population over longer time frames.

CDV had a more complicated relationship between fox density and vaccination success, as illustrated by large IQRs and extreme median values, reflecting the wide variety of model outcomes (which were often bimodal). Depending on the density of hosts at high risk pathogen introduction sites, a combination of random and firewall vaccination close to the anticipated introduction site may be advisable across a management area. For example, the remote southern beaches of SCI that are not frequently monitored and have a relatively low density of foxes may be a good site for random vaccination. The town where the harbor and airport are located, along with a very high density of foxes that are frequently monitored, may be a good site for a firewall or clustered vaccination strategy. In addition, higher levels of vaccination may be required for successful prevention of a CDV epidemic regardless of fox density, due to CDV’s longer infectious period resulting in faster pathogen transmission and more infected foxes [2].

A key assumption of our model to bear in mind when interpreting these findings is that island foxes are isolated from other host species which could also propagate an epidemic. Higher vaccination levels will likely be needed to protect mainland host populations that are frequently exposed to rabies or CDV by unvaccinated reservoir species [66–68]. We also assumed that all vaccinated foxes were fully protected for the one-year simulation. In order to achieve this, managers may have to account for vaccine failure by vaccinating more animals than they ultimately hope to have protected in the face of an epidemic. This level of vaccination may not be feasible depending on the cost of vaccines and the ease of animal capture for injectable vaccination. Alternative vaccination methods, such as oral vaccinations baits, could decrease the logistical burden of vaccination and allow managers to vaccinate animals over wider areas and in places which may be difficult for staff to reach by foot.

To be effective in preventing disease outbreak, there must be a high proportion of hosts vaccinated within the firewall and the firewall must be wider than the distance an animal will travel [16]. Island foxes have been recorded moving several kilometers during dispersal or temporary forays outside their home range [32,69], meaning that a successful vaccination firewall must be at least several kilometers wide to prevent an infected fox from traveling across it. The probability of vaccine saturation within a firewall is likely unattainable for large areas of SCI because of the high density of foxes in some areas of the island [31,70] and because vaccination is currently limited to hand injections due to the lack of approved oral rabies vaccines in California [30,71]. Even though this model assumed an unrealistic 100% vaccine saturation within firewalls, firewall vaccination was only substantially better than random vaccination when placed very near to pathogen introduction sites. However, even if a firewall does not stop an epidemic from spreading through the larger host population, managers should consider another potential benefit of clustering vaccinations: surviving, vaccinated animals will be able to maintain their social structure and relationships with known neighbors, potentially facilitating repopulation once the epidemic has passed.

There were substantial differences between the efficacy of firewalls located near vs. far from pathogen introduction site for both pathogens. A firewall strategy creates a small spatial zone where a disease can spread locally but will deterministically fade out due to the eventual depletion of susceptible hosts. However, as the number of infected animals increases, it becomes more likely that an infectious individual will cross the vaccine firewall and ignite the epidemic on the other side. This possibility is exacerbated when there is a high density of susceptible hosts (and hence R_0_>>1) on the infected side of a vaccine firewall. This scenario could be observed in our model simulations of CDV. CDV has a long infectious period, which resulted in a large number of simulated foxes in the infectious class at the same time, especially at high fox densities or when the firewall was placed far from the site of pathogen introduction. This large number of simultaneously infectious animals increased the chance that an infectious animal would cross the vaccination firewall, resulting in many model iterations where CDV jumped the firewall and the epidemic continued to spread through the entire fox population. However, when the firewall was placed closer to the site of pathogen introduction, fewer unvaccinated foxes were available to become infected and there was a much greater chance of pathogen fadeout with minimal population impacts.

Our model did not simulate contact rates between mates or family members, only non-related neighbors. Field data from island fox proximity collars showed that mated pairs and family members have higher home range overlap and contact rates than non-related pairs [31,39]. Including these close relationships would add variation to the overlap and contact rates simulated in the current model. Since the model predicts high transmission probability between non-related neighbors, it is reasonable to infer that related pairs will have higher transmission probabilities, which could accelerate the rate of pathogen spread in all model treatments. Previous CDV models and laboratory studies have also found higher transmission rates between individuals that were in contact frequently or for long periods of time [46,51,72]. Knowledge of fox social structure could help managers target highly connected individuals for vaccination in order to maximize the herd immunity benefit of vaccination (ex. adult females with pups, or family groups with yearling offspring assisting with raising the next generation of siblings).

The primary lesson from our research is that the potential impact of a pathogen on host populations and the effectiveness of monitoring and management strategies are heavily influenced by spatial variation in host density. Early detection of an epidemic is most likely if frequent monitoring is focused in areas with both a high risk of pathogen invasion and high population density. Vaccination programs in regions with high host density may not be as effective as providing high vaccine coverage in surrounding low-density areas where epidemic fadeout is more likely. Similarly, if pathogens are most likely to invade into low-density regions, vaccinating randomly throughout the area may be more effective. Previous studies have suggested that vaccination programs should also consider landscape features when designing deployment strategies: using natural barriers to enhance vaccination firewalls [73] and accounting for habitat corridors and roads that promote host movement [11,74]. Firewall vaccination would likely be most beneficial in situations where host populations living in and around key landscape features (such as narrow habitat corridors) could be saturated with vaccinations in order to more effectively reduced the number of hosts infected early in the epidemic and the risk of infection crossing the firewall. The influence of pathogen entry location suggests another possible ecological management tool—using landscape features to direct pathogen entry points away from areas with high hosts densities or towards regions with existing monitoring and vaccination programs [9].

Introduced and emerging diseases are becoming a common threat to wildlife populations as hosts and pathogens mix in previously unprecedented ways due to anthropogenic movement of animals, habitat loss and climate change altering host and vector geographic distributions, and the expansion of the interface between wildlife, domestic animals, and humans [75–77]. Here, we test two of the most common tools available for managing wildlife disease to help wildlife managers make strategic and precise decisions about how to utilize their limited conservation resources.

## Supporting information

S1 TableParameter values used to model the spread of rabies and canine distemper virus (CDV) in island foxes on San Clemente Island, California.MDS = maritime desert scrub. *N* ($\bar{x}$, variance) = normal distribution from which parameter value was sampled.(DOCX)

S2 Table Results of a spatially explicit disease model simulating the introduction of rabies into a population of San Clemente Island foxes with varying levels of sentinel monitoring. Day of epidemic detection was calculated as the day from the start of the simulation until the first or fifth unvaccinated, radio-collared sentinel animal died of disease. The percentage of the total fox population infected (i.e. latent, infectious, or dead) on the day of epidemic detection was used to assess the extent of pathogen spread.
(DOCX)

S3 Table Results of a spatially explicit disease model simulating the introduction of canine distemper into a population of San Clemente Island foxes with varying levels of sentinel monitoring. Day of epidemic detection was calculated as the day from the start of the simulation until the first or fifth unvaccinated, radio-collared sentinel animal died of disease. The percentage of the total fox population infected (i.e. latent, infectious, or dead) on the day of epidemic detection was used to assess the extent of pathogen spread.
(DOCX)

S4 Table Results of a spatially explicit disease model simulating the introduction of rabies into a population of San Clemente Island foxes with different vaccine distributions and percentages of the fox population vaccinated. The probability of epidemic fadeout was measured as the percentage of model iterations which resulted in no foxes remaining in the latent or infectious disease classes, so that rabies was extirpated from the fox population. The percentage of the total fox population infected (i.e. latent, infectious, or dead) at the end of the simulation (day 365) was used to assess the extent of pathogen spread. Simulations with firewall vaccination had either 5% of foxes (“near firewall”) or 20% of foxes (“far firewall”) on the side of the firewall where rabies was introduced.
(DOCX)

S5 Table Results of a spatially explicit disease model simulating the introduction of canine distemper into a population of San Clemente Island foxes with different vaccine distributions and percentages of the fox population vaccinated. The probability of epidemic fadeout was measured as the percentage of model iterations which resulted in no foxes remaining in the latent or infectious disease classes, so that canine distemper was extirpated from the fox population. The percentage of the total fox population infected (i.e. latent, infectious, or dead) at the end of the simulation (day 365) was used to assess the extent of pathogen spread. Simulations with firewall vaccination had either 5% of foxes (“near firewall”) or 20% of foxes (“far firewall”) on the side of the firewall where canine distemper was introduced.
(DOCX)

S1 Fig Empirical cumulative distribution curves representing the percentage of the total fox population infected (i.e. latent, infectious, or dead) one year after the introduction of rabies to an area of high (A, C, E) or low (B, D, F) fox density with random (A, B) or firewall (C-F) vaccination of 0, 10%, 30%, or 50% of the fox population. Simulations with firewall vaccination had either 5% of foxes (“near firewall”; C-D) or 20% of foxes (“far firewall”; E-F) on the side of the firewall where rabies was introduced. Curves represent the cumulative proportion of model iterations that resulted in a percentage of the total fox population infected (i.e. latent, infectious, or dead) which was less than or equal to the value of the x-axis. Squares represent median values. Dashed horizontal lines represent 25% and 75% quartiles.
(TIFF)

S2 Fig Empirical cumulative distribution curves representing the percentage of the total fox population infected (i.e. latent, infectious, or dead) one year after the introduction of canine distemper to an area of high (A, C, E) or low (B, D, F) fox density with random (A, B) or firewall (C-F) vaccination of 0, 10%, 30%, or 50% of the fox population. Simulations with firewall vaccination had either 5% of foxes (“near firewall”; C-D) or 20% of foxes (“far firewall”; E-F) on the side of the firewall where canine distemper was introduced. Curves represent the cumulative proportion of model iterations that resulted in a percentage of the total fox population infected (i.e. latent, infectious, or dead) which was less than or equal to the value of the x-axis. Squares represent median values. Dashed horizontal lines represent 25% and 75% quartiles.
(TIFF)
