# Supplementary material for: Vaccination and monitoring strategies for epidemic prevention and detection in the Channel Island fox (Urocyon littoralis)
Source: PLoS One. 2020 May 18;15(5):e0232705. doi: 10.1371/journal.pone.0232705 (PMC7233584; doi:10.1371/journal.pone.0232705)
Supplement: S1 Table — MDS = maritime desert scrub. N (x¯, variance) = normal distribution from which parameter value was sampled. (DOCX) [file pone.0232705.s001.docx]

**S1 Table. Parameter values used to model the spread of rabies and canine distemper virus (CDV) in island foxes on San Clemente Island, California.** MDS = maritime desert scrub. *N* (****, variance) = normal distribution from which parameter value was sampled.

| **Parameter** | **Value** | **Source** |
| --- | --- | --- |
| Home range radius  (meters; based on fox density) | High density: *N* (252.31, 2.28)  Medium-high density: *N* (282.09, 1.56)  Medium-low density: *N* (504.63, 2.02)  Low density: *N* (713.65, 1.59) | Field data [31] |
| Proportion of  home range overlap | ≤ 0.75 | Field data (maximum overlap observed between non-related pairs) [31] |
| Contact rate | Rabies (number of contacts/day)*: N* (0.02+0.88*m*, 0+0.25*m*)  CDV (seconds in contact/day)*: N* (1.42+58.57*m*, 0+2594.03*m*)  *m* = home range overlap | Field data [31] |
| Transmissibility | Rabies: 0.49*c* CDV: $N(\frac{1}{1+e^{-\left( 0.12+0.02c \right)}}, \frac{1}{e^{-(0.42c)}})$  *c* = contact rate | [16,51] |
| Transition probabilities^1^ | Rabies: latent to infectious class: 1/42 (max 90 days)  infectious to dead class: 1/4 (max 14 days)  CDV: latent to infectious class: 1/5 (max 14 days)  infectious to dead class: 1/21 (max 60 days) | [16,35,42–50] |
| Background  transmission rate^2^ | $1-{(1-f )}^{(\# of infectious foxes)}$  *f* = 0.000001 = probability per time-step of pathogen  transmission between two foxes with non-overlapping home ranges | Reed-Frost equation [41] |

^1^ Transition probabilities calculated as the average number of days a fox was in each disease class.

^2^ During the 206-day field study [31], during which foxes were radio-collared and their locations monitored, only one fox out of 40 made a foray outside of its study site. The probability of transmission between a focal fox and a fox on a foray was set to the arbitrarily low value of 1% because contact rates among foxes outside their home ranges are not known. The resulting daily risk each focal fox had of encountering another fox during a foray outside its home range and contracting a pathogen (if that other fox was infectious) was approximately $(1\div40\div206)\times0.01\approx0.000001$.
