# Supplementary material for: Vaccination and monitoring strategies for epidemic prevention and detection in the Channel Island fox (Urocyon littoralis)
Source: PLoS One. 2020 May 18;15(5):e0232705. doi: 10.1371/journal.pone.0232705 (PMC7233584; doi:10.1371/journal.pone.0232705)
Supplement: S2 Table — Day of epidemic detection was calculated as the day from the start of the simulation until the first or fifth unvaccinated, radio-collared sentinel animal died of disease. The percentage of the total fox population infected (i.e. latent, infectious, or dead) on the day of epidemic detection was used to assess the extent of pathogen spread. (DOCX) [file pone.0232705.s002.docx]

**S2 Table. Results of a spatially explicit disease model simulating the introduction of rabies into a population of San Clemente Island foxes with varying levels of sentinel monitoring.** Day of epidemic detection was calculated as the day from the start of the simulation until the first or fifth unvaccinated, radio-collared sentinel animal died of disease. The percentage of the total fox population infected (i.e. latent, infectious, or dead) on the day of epidemic detection was used to assess the extent of pathogen spread.

| **Density of foxes at the site of pathogen introduction** | **Number of sentinels** | **Median day of epidemic detection (Q1, Q3)** | | **Median percentage of foxes infected on day of detection (Q1, Q3)** | |
| --- | --- | --- | --- | --- | --- |
|  |  | 1st sentinel mortality | 5th sentinel mortality | 1st sentinel mortality | 5th sentinel mortality |
| High-density | 50 | 16 (27, 7) | 93 (69, 120) | 0.47 (0.22, 0.97) | 3.85 (9.31, 0.91) |
|  | 75 | 9 (17, 4) | 59 (45, 78) | 0.39 (0.20, 0.71) | 1.78 (4.88, 0.48) |
|  | 100 | 7 (13, 3) | 45 (32, 61) | 0.34 (0.20, 0.63) | 1.26 (2.85, 0.42) |
|  | 125 | 6 (10, 3) | 36 (27, 47) | 0.32 (0.19, 0.57) | 0.94 (2.29, 0.34) |
|  | 150 | 5 (9, 2) | 31 (23, 40) | 0.30 (0.12, 0.52) | 0.80 (1.97, 0.33) |
| Low-density | 50 | 14 (6, 26) | 93 (68, 122) | 0.33 (0.55, 0.20) | 0.99 (1.96, 0.39) |
|  | 75 | 9 (4, 17) | 63 (46, 85) | 0.30 (0.48, 0.19) | 0.69 (1.47, 0.30) |
|  | 100 | 7 (3, 14) | 45 (35, 61) | 0.29 (0.43, 0.13) | 0.57 (1.08, 0.27) |
|  | 125 | 6 (3, 11) | 37 (28, 49) | 0.29 (0.40, 0.11) | 0.50 (0.96, 0.22) |
|  | 150 | 5 (2, 9) | 31 (24, 41) | 0.28 (0.40, 0.11) | 0.46 (0.86, 0.22) |
