# Supplementary material for: Vaccination and monitoring strategies for epidemic prevention and detection in the Channel Island fox (Urocyon littoralis)
Source: PLoS One. 2020 May 18;15(5):e0232705. doi: 10.1371/journal.pone.0232705 (PMC7233584; doi:10.1371/journal.pone.0232705)
Supplement: S5 Table — The probability of epidemic fadeout was measured as the percentage of model iterations which resulted in no foxes remaining in the latent or infectious disease classes, so that canine distemper was extirpated from the fox population. The percentage of the total fox population infected (i.e. latent, infectious, or dead) at the end of the simulation (day 365) was used to assess the extent of pathogen spread. Simulations with firewall vaccination had either 5% of foxes (“near firewall”) or 20% of foxes (“far firewall”) on the side of the firewall where canine distemper was introduced. (DOCX) [file pone.0232705.s005.docx]

**S5 Table. Results of a spatially explicit disease model simulating the introduction of canine distemper into a population of San Clemente Island foxes with different vaccine distributions and percentages of the fox population vaccinated.** The probability of epidemic fadeout was measured as the percentage of model iterations which resulted in no foxes remaining in the latent or infectious disease classes, so that canine distemper was extirpated from the fox population. The percentage of the total fox population infected (i.e. latent, infectious, or dead) at the end of the simulation (day 365) was used to assess the extent of pathogen spread. Simulations with firewall vaccination had either 5% of foxes (“near firewall”) or 20% of foxes (“far firewall”) on the side of the firewall where canine distemper was introduced.

| **Density of foxes at the site of pathogen introduction** | **Vaccination distribution strategy** | **Percentage of foxes vaccinated** | **Probability of epidemic fadeout ± binomial SE** | **Median percentage of foxes infected on day 365 (Q1, Q3)** |
| --- | --- | --- | --- | --- |
| High-density | None | 0% | 99.0 +/- 0.3 | 95.34 (93.38, 96.60) |
|  | Random | 10% | 97.5 +/- 0.3 | 80.89 (74.94, 83.98) |
|  |  | 30% | 98.5 +/- 0.3 | 40.03 (30.03, 47.13) |
|  |  | 50% | 100.0 +/- 0.3 | 5.29 (0.57, 14.77) |
|  | Near firewall | 10% | 97.8 +/- 0.3 | 4.04 (1.77, 82.85) |
|  |  | 30% | 98.8 +/- 0.3 | 3.95 (2.05, 59.21) |
|  |  | 50% | 99.0 +/- 0.3 | 3.38 (1.82, 5.28) |
|  | Far firewall | 10% | 97.5 +/- 0.3 | 84.87 (81.54, 86.44) |
|  |  | 30% | 96.1 +/- 0.3 | 64.53 (55.08, 66.45) |
|  |  | 50% | 99.5 +/- 0.3 | 42.73 (20.49, 46.38) |
| Low-density | None | 0% | 97.9 +/- 0.3 | 94.74 (87.72, 96.25) |
|  | Random | 10% | 96.0 +/- 0.3 | 77.31 (3.31, 83.1) |
|  |  | 30% | 98.7 +/- 0.3 | 1.86 (0.48, 6.98) |
|  |  | 50% | 100.0+/- 0.3 | 0.41 (0.19, 1.01) |
|  | Near firewall | 10% | 98.7 +/- 0.3 | 5.05 (4.05, 84.23) |
|  |  | 30% | 99.8 +/- 0.3 | 4.94 (4.25, 66.18) |
|  |  | 50% | 99.9 +/- 0.3 | 4.69 (3.96, 47.97) |
|  | Far firewall | 10% | 98.1 +/- 0.3 | 83.5 (21.50, 85.75) |
|  |  | 30% | 99.1 +/- 0.3 | 64.97 (5.45, 67.52) |
|  |  | 50% | 99.6 +/- 0.3 | 41.46 (13.20, 47.41) |
