# Supplementary figures and images for: Vaccination and monitoring strategies for epidemic prevention and detection in the Channel Island fox (Urocyon littoralis)
Source: PLoS One. 2020 May 18;15(5):e0232705. doi: 10.1371/journal.pone.0232705 (PMC7233584; doi:10.1371/journal.pone.0232705)

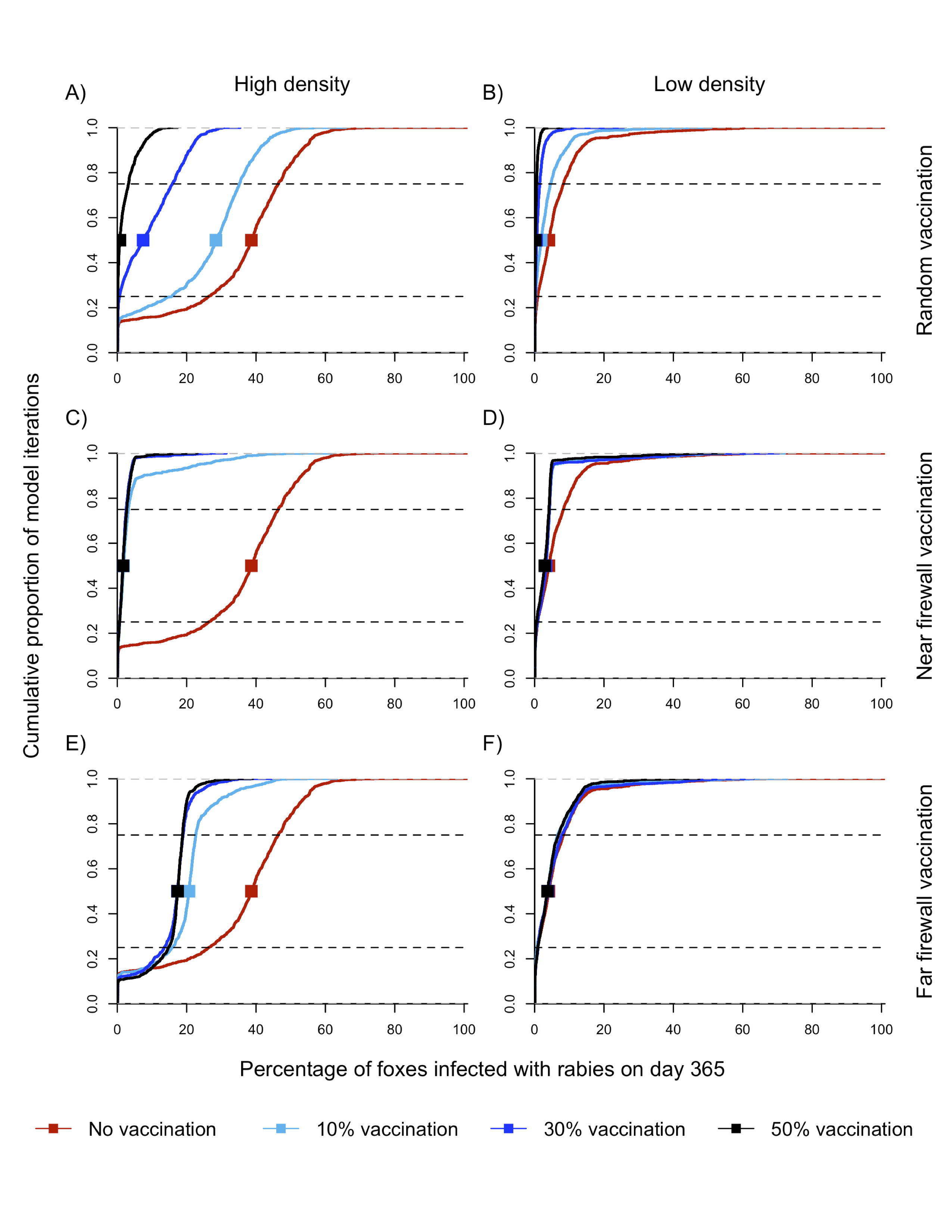

Supplement: S1 Fig — Empirical cumulative distribution curves representing the percentage of the total fox population infected (i.e. latent, infectious, or dead) one year after the introduction of rabies to an area of high (A, C, E) or low (B, D, F) fox density with random (A, B) or firewall (C-F) vaccination of 0, 10%, 30%, or 50% of the fox population. Simulations with firewall vaccination had either 5% of foxes (“near firewall”; C-D) or 20% of foxes (“far firewall”; E-F) on the side of the firewall where rabies was introduced. Curves represent the cumulative proportion of model iterations that resulted in a percentage of the total fox population infected (i.e. latent, infectious, or dead) which was less than or equal to the value of the x-axis. Squares represent median values. Dashed horizontal lines represent 25% and 75% quartiles. (TIFF) [file pone.0232705.s006.tiff]

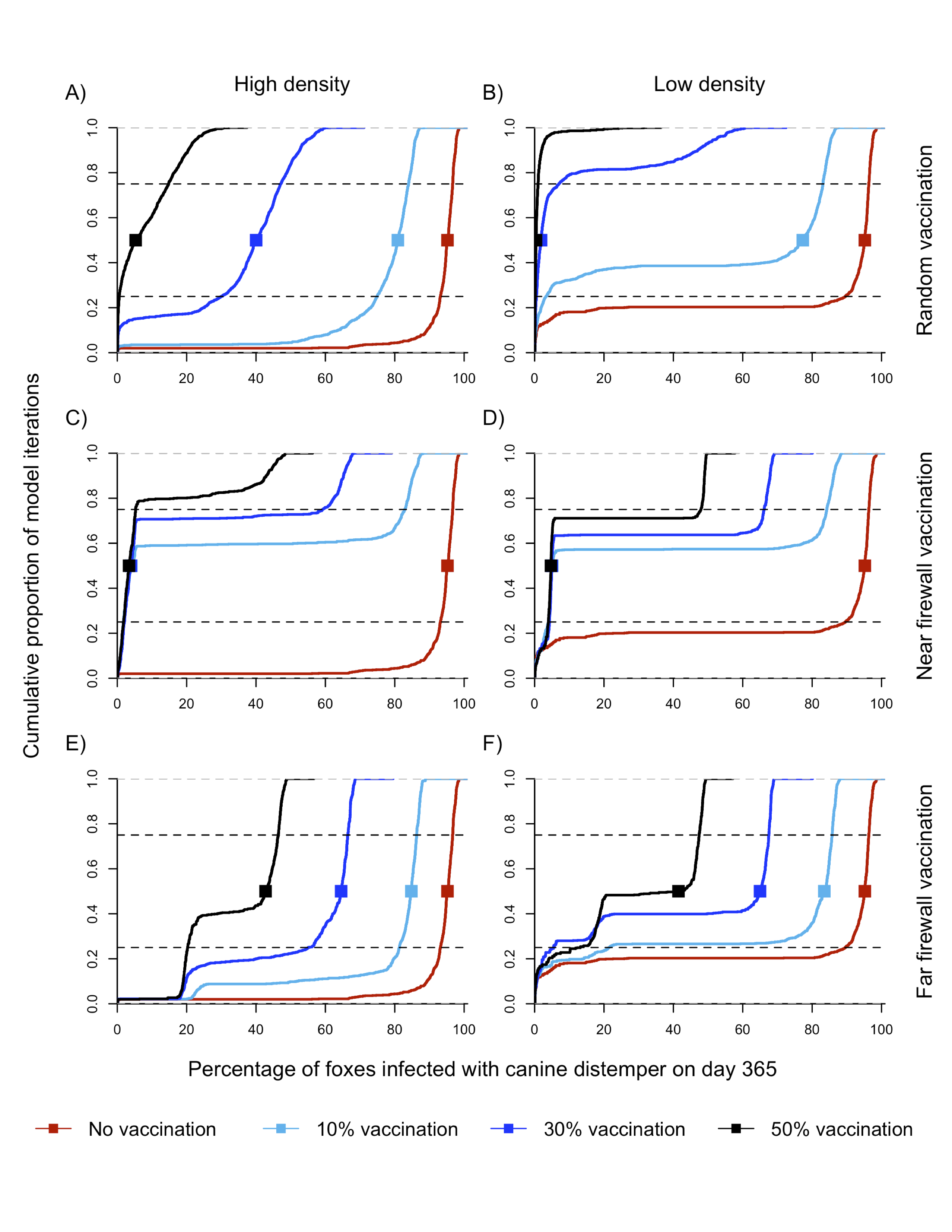

Supplement: S2 Fig — Empirical cumulative distribution curves representing the percentage of the total fox population infected (i.e. latent, infectious, or dead) one year after the introduction of canine distemper to an area of high (A, C, E) or low (B, D, F) fox density with random (A, B) or firewall (C-F) vaccination of 0, 10%, 30%, or 50% of the fox population. Simulations with firewall vaccination had either 5% of foxes (“near firewall”; C-D) or 20% of foxes (“far firewall”; E-F) on the side of the firewall where canine distemper was introduced. Curves represent the cumulative proportion of model iterations that resulted in a percentage of the total fox population infected (i.e. latent, infectious, or dead) which was less than or equal to the value of the x-axis. Squares represent median values. Dashed horizontal lines represent 25% and 75% quartiles. (TIFF) [file pone.0232705.s007.tiff]
